# Performance Evaluation and Validation of Air Samplers To Detect Aerosolized Coxiella burnetii

**DOI:** 10.1128/spectrum.00655-22

**Published:** 2022-09-08

**Authors:** A. M. Hasanthi Abeykoon, Megan Poon, Simon M. Firestone, Mark A. Stevenson, Anke K. Wiethoelter, Gemma A. Vincent

**Affiliations:** a Faculty of Veterinary and Agricultural Sciences, University of Melbourne, Parkville, Victoria, Australia; b Australian Rickettsial Reference Laboratory, University Hospital Geelong, Geelong, Victoria, Australia; University of California, Davis

**Keywords:** *Coxiella burnetii*, air sampling, airborne microorganisms

## Abstract

Coxiella burnetii, the etiological agent of Q fever, is an intracellular zoonotic pathogen transmitted via the respiratory route. Once released from infected animals, C. burnetii can travel long distances through air before infecting another host. As such, the ability to detect the presence of C. burnetii in air is important. In this study, three air samplers, AirPort MD8, BioSampler, and the Coriolis Micro, were assessed against a set of predetermined criteria in the presence of three different aerosolized C. burnetii concentrations. Two liquid collection media, phosphate-buffered saline (PBS) and alkaline polyethylene glycol (Alk PEG), were tested with devices requiring a collection liquid. Samples were tested by quantitative polymerase chain reaction assay (qPCR) targeting the single-copy *com1* gene or multicopy insertion element IS*1111*. All air samplers performed well at detecting airborne C. burnetii across the range of concentrations tested. At high nebulized concentrations, AirPort MD8 showed higher, but variable, recovery probabilities. While the BioSampler and Coriolis Micro recovered C. burnetii at lower concentrations, the replicates were far more repeatable. At low and intermediate nebulized concentrations, results were comparable in the trials between air samplers, although the AirPort MD8 had consistently higher recovery probabilities. In this first study validating air samplers for their ability to detect aerosolized C. burnetii, we found that while all samplers performed well, not all samplers were equal. It is important that these results are further validated under field conditions. These findings will further inform efforts to detect airborne C. burnetii around known point sources of infection.

**IMPORTANCE** Coxiella burnetii causes Q fever in humans and coxiellosis in animals. It is important to know if C. burnetii is present in the air around putative sources as it is transmitted via inhalation. This study assessed air samplers (AirPort MD8, BioSampler, and Coriolis Micro) for their efficacy in detecting C. burnetii. Our results show that all three devices could detect aerosolized bacteria effectively; however, at high concentrations the AirPort performed better than the other two devices, showing higher percent recovery. At intermediate and low concentrations AirPort detected at a level higher than or similar to that of other samplers. Quantification of samples was hindered by the limit of quantitation of the qPCR assay. Compared with the other two devices, the AirPort was easier to handle and clean in the field. Testing air around likely sources (e.g., farms, abattoirs, and livestock saleyards) using validated sampling devices will help better estimate the risk of Q fever to nearby communities.

## INTRODUCTION

Coxiella burnetii causes Q fever in humans. This zoonotic disease has acute and chronic forms ranging from mild flu-like illness to fatal endocarditis (1, 2). Although many wild and domestic animals can be infected with C. burnetii without clinical signs (3), this agent can cause loss in milk production and abortions in domestic ruminants (4). Infected cattle, sheep, and goats shed C. burnetii through milk, feces, urine, birth products, and aborted materials with concentrations as high as billions of organisms per gram of placenta detected (5, 6). Once in the environment, C. burnetii persists by converting into a spore-like small cell variant (SCV) form until it enters another host (3, 7). Coxiella burnetii has been estimated to have an infectious dose of around one organism (8, 9) and, due to the possibility of large-scale production, has also been listed as a potential bioterrorism agent by the Centers for Disease Control and Prevention (CDC) (10, 11). These unique characteristics and its airborne transmission capability over long distances make Q fever an emerging public health concern in many parts of the world.

Several large Q fever outbreaks to date have confirmed close associations between Q fever incidence and airborne transmission (12–15). Coxiella burnetii excreted by infected domestic ruminants and reaerosolized from secondary contaminants or surfaces such as deposited dust, soil, manure, bedding, aborted material, and infected carcasses may travel far from the original point of release before infecting another host. From farms that are likely sources of infection, the highest risk has been estimated to be up to 4 km away (16, 17). Human infection may even have occurred beyond 10 km from an infected farm (18), although the outbreak investigation did not include environmental testing outside the farm to confirm C. burnetii contamination nor did investigators rule out the possibility that all cases residing greater than 10 km away had not visited areas closer to the infected farm at some point in time (18). Accurate testing, detection, and quantification of C. burnetii in air are important to confidently assess the geographic dispersal and infection risk around a putative source (19).

Coxiella burnetii is reported to be abundantly present in air during periods such as lambing/kidding or shearing as large amounts of organisms are released into the environment within a short period of time (20). Several studies have detected circulating C. burnetii using a variety of air sampling devices, with quantitation performed in some cases (15, 21–36). To the best of our knowledge, none of these studies involved attempts at standardization or validation of air sampling devices, so the rationale for the choice of sampler in each study is unclear (37). Given the unique nature of this bacterium, it is important to use validated air sampling methods to better understand the risk arising from C. burnetii in aerosols and inform Q fever detection, management planning, and preventive strategies. This study aims to compare and validate three air samplers, each with different modes of function, on their ability to qualitatively and quantitatively detect aerosolized C. burnetii. Given that C. burnetii is an emerging zoonotic airborne pathogen and a potential bioterrorism agent, this study is timely.

## RESULTS

### LoD, LoQ, and extraction efficiency of the C. burnetii *com1* qPCR.

**FIG 1:**
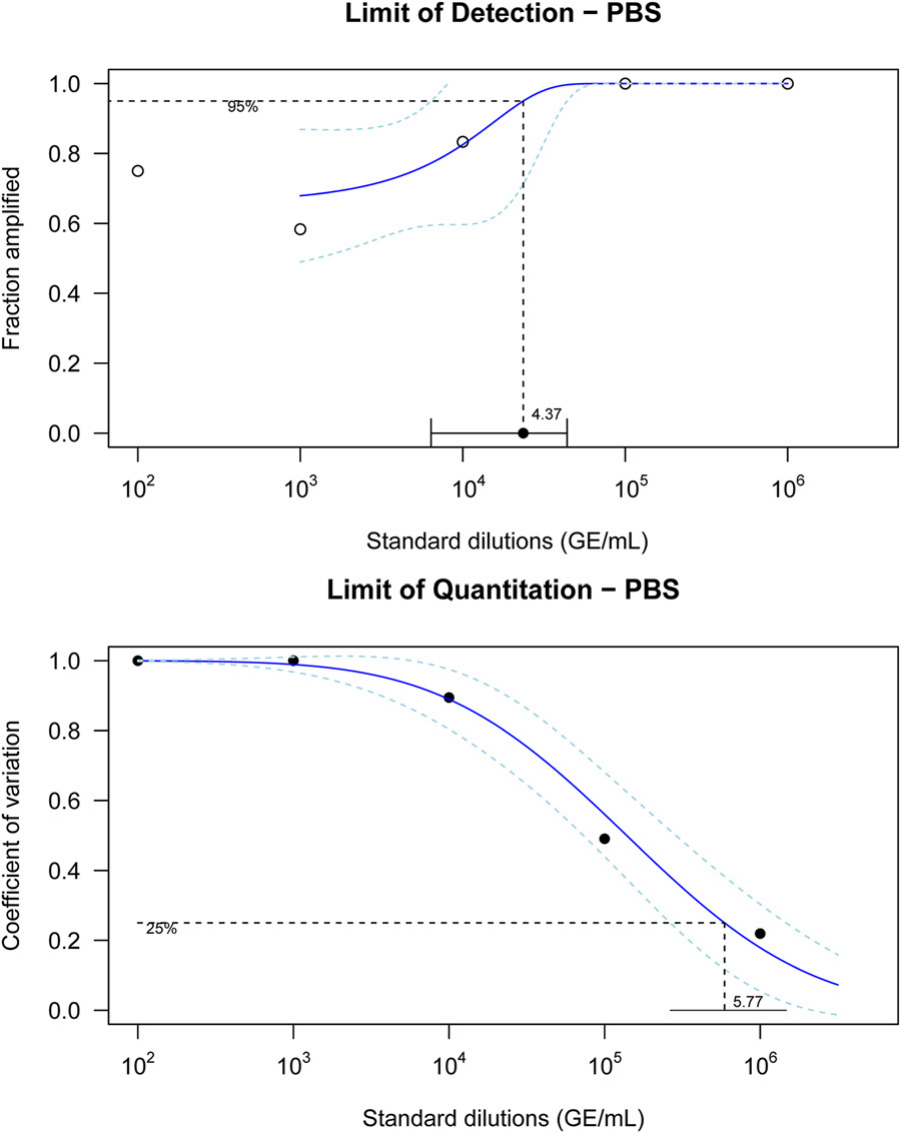
Limit of detection (top) and limit of quantitation (bottom) estimates of phosphate-buffered saline and their 95% confidence intervals. Solid lines show lines of best fit, while the two dashed lines above and below show their 95% confidence intervals.

The limit of detection (LoD), the limit of quantitation (LoQ), and the extraction efficiency for three substrates are presented in Table 1. In summary, the LoDs, LoQs, and extraction efficiencies were similar across substrates. The lowest LoD of the *com1* quantitative PCR (qPCR) assay was observed with phosphate-buffered saline (PBS) while the lowest LoQ was observed with alkaline polyethylene glycol (Alk PEG). The extraction efficiency was the highest when dissolved gelatin membrane filter (GMF) was used. Line plots showing the fraction of replicates amplified and coefficient of variation (CV) as a function of dilution (genomic equivalents [GE] per milliliter) for LoD and LoQ for PBS are shown in Fig. 1, top and bottom, respectively. Similar plots for the other substrates are provided in the supplemental material (Fig. S1 and S2).

**TABLE 1:** Limits of detection, limits of quantitation, and extraction efficiencies of three substrates^*a*^

Substrate	LoD, GE/mL (95% CI)	LoQ, GE/mL (95% CI)	Extraction efficiency (%)
PBS	10^4.37^ (10^3.80^–10^4.64^)	10^5.77^ (10^5.42^–10^6.17^)	30.0
Alk PEG	10^4.60^ (10^4.00^–10^4.84^)	10^5.56^ (10^5.26^–10^5.90^)	25.8
Dissolved GMF	10^4.43^ (10^3.93^–10^4.65^)	10^5.67^ (10^5.14^–10^6.24^)	36.3

a PBS, phosphate-buffered saline; Alk PEG, alkaline polyethylene glycol; GMF, gelatin membrane filter; 95% CI, 95% confidence interval.

### Air sampler trials.

**FIG 2:**
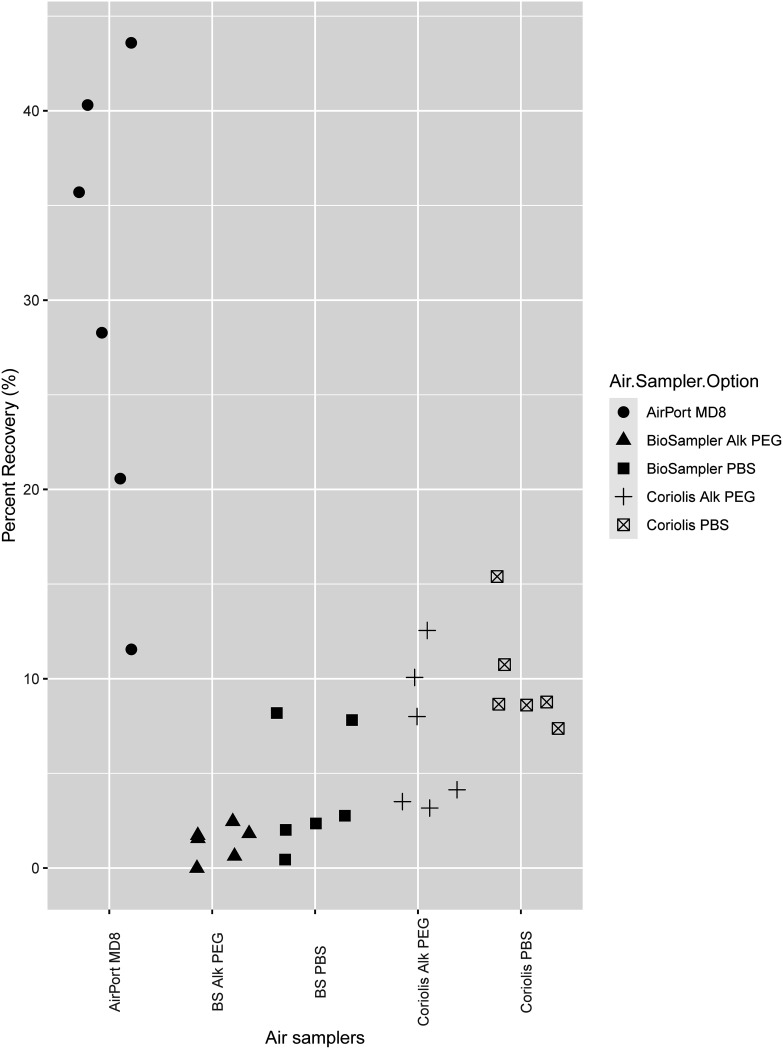
Percent recoveries (percentage of total genomic equivalent [GE] recovered over total GE nebulized) when the high Coxiella burnetii concentration (10^6^ GE/mL) was nebulized. Quantification is based on the *com1* qPCR assay. PBS, phosphate-buffered saline; Alk PEG, alkaline polyethylene glycol.

Results from the high-nebulized-concentration trials are shown in Fig. 2 and Table 2 (see supplemental material for detailed results). Only samples collected by the AirPort MD8 resulted in concentrations that could be accurately quantified based on the LoQ. There were lower precision and a lower degree of confidence in the percent recoveries for the other air sampler options. The AirPort MD8 had the highest percent recovery and the highest mean GE recovered per liter of air. However, the percent recoveries were more variable among the six replicates of AirPort MD8 than in the other devices. The BioSampler using Alk PEG as the collection medium had the lowest percent recovery and mean GE per liter of air.

**TABLE 2:** Recovered concentrations of C. burnetii in high-nebulized-concentration (10^6^ GE/mL) trial^*a*^

Air sampler	Mean recovered concentration	
	In collected sample (GE/mL)	Per L of air (GE/L)
AirPort MD8	10^5.66^	10^3.26^
BioSampler PBS	10^4.59^	10^2.77^
BioSampler Alk PEG	10^4.06^	10^2.25^
Coriolis PBS	10^4.34^	10^2.61^
Coriolis Alk PEG	10^4.18^	10^2.41^

a Quantification is based on the *com1* qPCR assay. PBS, phosphate-buffered saline; Alk PEG, alkaline polyethylene glycol.

Results for the low- and intermediate-concentration trials, in which samples were tested with both *com1* and IS*1111* PCR assays, are shown in Table 3. Recovered concentrations of C. burnetii were below the quantitation limit of the *com1* assay; therefore, results are presented as the number of trials (out of six) in which a positive result was returned.

**TABLE 3:** Qualitative results of low- and intermediate-concentration trials based on *com1* and IS*1111* qPCR assays

Air sampler^*a*^	No. of positive trials/total no. of trials for nebulized concn:			
	Low (10^3^ GE/mL)		Intermediate (10^4^ GE/mL)	
	*com1*	IS*1111*	*com1*	IS*1111*
AirPort MD8	0/6	4/6	4/6	6/6
BioSampler PBS	0/6	0/6	3/6	6/6
BioSampler Alk PEG	0/6	3/6	1/6	4/6
Coriolis PBS	0/6	1/6	0/6	6/6
Coriolis Alk PEG	0/6	3/6	2/6	5/6

a PBS, phosphate-buffered saline; Alk PEG, alkaline polyethylene glycol.

The greatest number of trials where ≥1 of the PCR replicates was detected as positive was obtained using the AirPort MD8. In the intermediate-concentration trial, almost all air sampler conditions showed high positivity with IS*1111*. The *com1* assay results, however, were variable, with AirPort MD8 showing the most detections. In the presence of low concentrations, three of the sampling conditions (AirPort MD8, BioSampler using Alk PEG, and Coriolis using Alk PEG) showed high positivity with IS*1111*. In the low-concentration trial, the concentrations of the collected as well as nebulized C. burnetii were below the LoD of the *com1* assay.

### Comments on air sampler characteristics.

Of the three air samplers tested in this study, the AirPort MD8 had the best operational characteristics for use in the field, in terms of ease of use and cleaning and no requirement for auxiliary equipment and power source. The AirPort MD8 was lighter and had a moderate flow rate with reasonable duration of battery power. Handling GMFs is more convenient than handling liquids in the field; however, they are relatively expensive compared with liquid collection media. The AirPort MD8 had limits for the volume of air that could be sampled during a single sampling run.

## DISCUSSION

To our knowledge, this is the first attempt to compare or validate air sampling methods (either qualitatively or quantitatively) for detecting aerosolized C. burnetii. All three samplers were sensitive enough to detect C. burnetii at intermediate concentrations. The AirPort MD8 performed the best across the range of C. burnetii concentrations tested and was the easiest to use and clean in the field. At the highest C. burnetii concentration tested, percent recovery for the AirPort MD8 was 11.6 to 43.6%, compared to 0 to 15.4% for the other two samplers tested. This finding is in line with previous studies (38); however, there was greater variability among the replicates of AirPort MD8 than in the other samplers, as also reported in previous studies of air sampling for other bacteria and fungi (39–41). The mechanism of the AirPort MD8, air filtration, has been shown to yield higher DNA amounts than liquid impingers, albeit in different contexts (42, 43). At the intermediate and low concentrations tested, all air samplers recovered nebulized C. burnetii to differing extents.

Both the LoD and LoQ presented in this study account for the whole method, including DNA extraction, and while quantitation values reported in the literature are lower than the LoQ of the current study (25, 26, 33), their assay validation details were not provided. Measured concentrations of all samples in the high-concentration trial were below the LoQ, except for two replicates in the AirPort MD8. The levels of confidence in quantitation values below the LoQ are low as reflected by increasing percent coefficients of variation (CV) (see the supplemental material for details). While the LoQ of the process hinders quantitative assessment when low concentrations are circulating, the relative significance of detection/nondetection is likely to be important in field situations due to the low infectious dose of C. burnetii; therefore, the presence of even a small number of organisms in the air is critical (9).

It is important to note that while a detection below the LoD may be a true positive, repeated testing of the same sample is expected to yield a positive result only <95% of the time (44). Criteria such as observing correct curve morphology and having valid PCR controls form part of the consideration of whether or not it is a genuine amplification (45).

**FIG 3:**
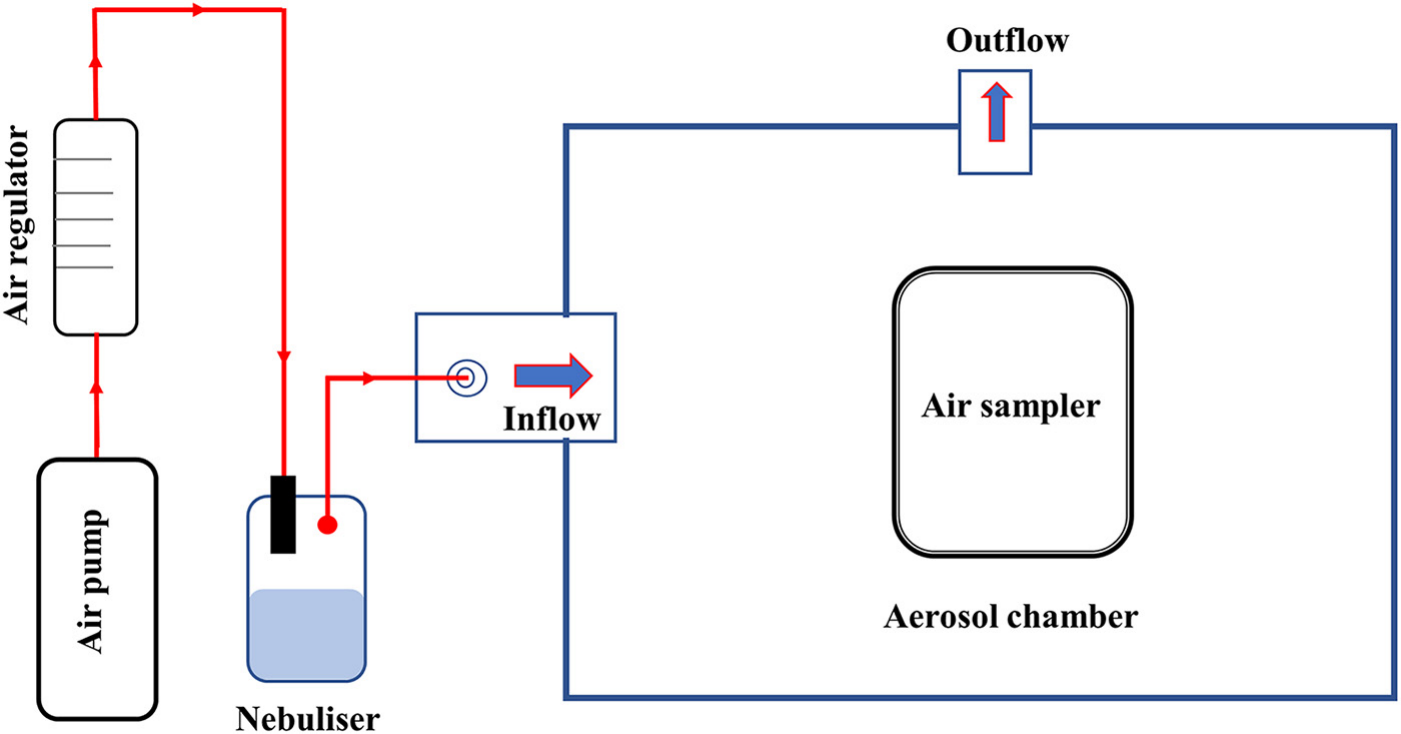
Diagram of the experimental unit.

Collection efficiency for small particles with liquid impingers may be lower when highly viscous collection liquids such as Alk PEG are used as the air current moving into the liquid slows down, resulting in reduced frequency of particle removal in the aerosol toward the inner wall of the collector (46, 47), and PBS has been favored as the collection liquid of choice for impingers by others (42). When using Alk PEG as the collection medium, slightly lower C. burnetii recoveries were observed with the BioSampler and Coriolis in the high- and intermediate-concentration trials (Fig. 3; see also Tables 2 and 3), but the two collection media can be recommended equally to be used in liquid impingers as PBS had a recovery only slightly increased over that for Alk PEG.

The AirPort MD8 was more user friendly in aspects important for operation in the field than the liquid impingers. This device uses a GMF that has shown higher collection efficiency than other types of filters (48) due to its layered structure, decreasing the chance of particles passing through. The AirPort MD8 also has moderate airflow rates that enable air sampling to be achieved within a reasonable time. However, for long-term or large-scale monitoring, the other two air samplers may be more suitable due to the limited sampling volume and duration of sampling of the AirPort MD8 and the relatively high cost of GMFs.

Several limitations to our study are recognized. Our experimental design assumed that the total volume (and the total number of GE) lost during nebulization was available for sampling. While this might not be the case, as some particles may get attached to the tubing or surfaces of the chamber and sampling devices, settle in, or escape the chamber, we assumed that these losses remain constant for all trials as the same trial conditions were maintained throughout.

Using PCR for detection of C. burnetii does not determine its viability and thus infectivity. However, detection of live organisms, particularly from environmental samples, is challenging and resource intensive due to the bacterium’s intracellular nature and the requirement for a biosafety level 3 laboratory for growth (49).

Assay validation was performed using synthetic DNA in which the number of copies of the PCR target sequence was calculated from the DNA concentration provided by the manufacturer. The use of whole C. burnetii organisms may have provided a more accurate determination of the LoD and LoQ; however, this would have required an independent assay with which to quantify a whole-cell C. burnetii preparation. DNA extraction from whole cells may have resulted in a lower extraction efficiency, but the shorter fragments of synthetic DNA may have bound to the DNA extraction columns less efficiently than larger, genomic DNA. Therefore, it is considered unlikely that the LoD and LoQ parameters determined would have been significantly different if whole-cell C. burnetii had been used for validation.

Axenically grown C. burnetii suspensions were nebulized in this study rather than bacteria that were treated or inactivated with the anticipation of mimicking natural conditions. However, organisms released into the environment from infected animals would rarely remain as standalone bacteria (0.2 to 0.5 μm in size) but rather aggregate or attach themselves to larger particles such as dust. In the light of this, it is possible that recovery probabilities in the field may be greater than observed in this study, as most air sampling devices demonstrate higher collection efficiencies for particles of >1 μm (42, 50, 51).

### Conclusions and recommendations.

Based on our findings and considering predefined criteria on deciding the ideal air sampler, we conclude that the AirPort MD8 was the best sampling device to detect C. burnetii of the three devices that were tested. However, if long-term air monitoring or large-scale air sampling for C. burnetii is aimed for in further research, one of the other air sampler options would appear a better choice. The test atmosphere in the laboratory is different from the field situation where inert particles such as dust to which C. burnetii may attach are present. Therefore, it is recommended to extend this validation study by performing field experiments.

## MATERIALS AND METHODS

Predefined criteria were developed, *a priori*, for comparing air samplers and considering which had the best overall performance characteristics for detecting circulating C. burnetii:

1. The ability to detect high or low concentrations of the C. burnetii in air (efficacy);
2. The time and duration of sampling; and
3. Air sampler properties including ease of handling (weight, size) and operation in the field (ease of use, auxiliary equipment, dependence on vacuum pumps, water, electricity, etc.) and ease of cleaning and disinfection.

These criteria were adapted from the ISO 14698-1 standard (52), which is a standard for principles and methods of biocontamination control in controlled environments, which was not the specific focus of the current study and, therefore, was not followed in detail subsequently.

### Sampling devices and collection media.

Three air sampling devices were evaluated in this study: (i) AirPort MD8 (Sartorius, Germany), (ii) BioSampler (SKC Inc., USA), and (iii) Coriolis Micro (Bertin Technologies, France). These air samplers were specifically chosen for this study as they were representative of air samplers on the market in terms of their modes of functions including flow rate (high, moderate, and low), user friendliness in the field, and ease of cleaning. Two options for liquid collection media tested in this study were PBS and Alk PEG, prepared using PEG 200 (Sigma-Aldrich) as described previously (53). Characteristics of these devices in terms of handling and operation in the field and general features including their modes of action are summarized in Table 4.

**TABLE 4:** Characteristics of air samplers

Air sampler	Mode of action	Flow rate	Operation					Other features
			Wt and size	Ease of use	Auxiliary equipment	Power	Ease of cleaning	
AirPort MD8	Filters air through and impacts onto a gelatin membrane filter (GMF) with 3-μm pores	Moderate, 30, 40, and 50 L/min	2.5 kg; 300 × 135 × 165 mm	High; one person can perform sampling; filters easy to handle	None	Battery that can last approx. 4.5 h at 50 L/min	High; only one part needs to be removed for cleaning/disinfection	Can preset sample vol; limited sampling vol and time per given run; GMFs are relatively expensive
BioSampler	A sonic flow is achieved which draws aerosols into a nozzle that is directed toward a swirling liquid collection medium	Low, set at 12.5 L/min	BioLite pump (SKC Inc., USA), 6.05 kg’ 162 × 209 × 338 mm	Low; requires at least two persons to perform sampling as the glass structure is fragile and the pump is heavy and to perform handling of liquid	High-vol vacuum pump	No batteries are available, and therefore, it needs a power source	Low; need to disassemble the glass sampler parts prior to cleaning/disinfection, which is challenging in the field if multiple samples are to be collected	Unlimited sampling vol and time per sample provided that the collection liquid is restored
Coriolis Micro	Air is drawn into a conical vial containing the liquid collection medium, forming a vortex	High, 100 to 300 L/min	3 kg; 220 × 330 × 360 mm; needs care when handling in the field due to its shape	Moderate; one person can perform sampling, but the plastic air intake and screw are fragile and need care when handling	None	Needs a power source if sampling for longer than an hour	Low; need to disassemble several plastic and metal parts of the air intake for cleaning/disinfection	Speedy sampling; longer sampling (up to 6 h) with the long-term monitoring (LTM) option; can be programmed to start with a delay

### Nebulization equipment.

Air samplers to be tested were placed within a custom-made, 150-L-capacity aerosol chamber, which was placed within a class II biological safety cabinet (BSC II). An air pump connected to an air regulator supplied controlled airflow at 10 L/min to a nebulizer (Collison 6-jet CN25 nebulizer; BGI Instruments). The nebulizer generated and supplied the aerosol (approximate particle size, 0.78 to 9 μm [54]) containing C. burnetii into the aerosol chamber. The air movement created by the nebulizer was sufficient to distribute aerosols inside the small capacity of the chamber. Prior to conducting the main study trials, three preliminary trials and a smoke test were performed to standardize trial conditions (details not presented in this paper). A schematic diagram of this unit is shown in Fig. 3.

### Preparation of Coxiella burnetii for nebulization.

Coxiella burnetii Nine Mile RSA439 (phase II, clone 4) was grown axenically in modified acidified citrate cysteine medium (ACCM-2; Sunrise Science Products, USA) for 7 days as previously described (55). The C. burnetii culture was quantified by qPCR (details below) and harvested by centrifugation at 3,000 × *g* for 15 min. Harvested cultures were stored at −80°C in freezing medium containing 10% fetal calf serum, 10% dimethyl sulfoxide (DMSO) in RPMI medium using a Mr. Frosty freezing container (Thermo Fisher Scientific) in 1.5-mL cryovials until used in the experiment. Suspensions of C. burnetii were prepared for each trial by dilution of a thawed aliquot to the required concentration in PBS. Once prepared, the suspension was stored at 4°C and used within 7 days.

### Trials.

In this study, 500 L of air was sampled with each air sampler, and the details of sampling conditions tested are shown in Table 5.

**TABLE 5:** Details of sampling condition of each air sampler^*a*^

Air sampler	Collection medium	Flow rate (L/min)	Sampling time (min)
AirPort MD8	GMF	50	10
BioSampler	Alk PEG	12.5	40
BioSampler	PBS	12.5	40
Coriolis Micro	Alk PEG	100	5
Coriolis Micro	PBS	100	5

a GMF, gelatin membrane filter; Alk PEG, alkaline polyethylene glycol; PBS, phosphate-buffered saline.

Three trials were performed by nebulizing low (10^3^ GE/mL)-, intermediate (10^4^ GE/mL)-, and high (10^6^ GE/mL)-C. burnetii-concentration suspensions. These concentrations were chosen based on the literature and observations during pilot field sampling on an infected dairy goat farm where concentrations up to 10^4.93^ GE/mL (10^2.23^ GE/L of air sampled) were detected when a large number of kidding events occurred (unpublished data). It has been reported in the literature that concentrations ranging from 10^−1.39^ to 10^0.47^ GE/L of air sampled have been detected inside sheep housing and concentrations ranging from 10^−0.64^ to 10^−2.56^ GE/L of air sampled have been detected outside (25, 26, 33). It is expected that circulating concentrations greater than 10^6^ GE/mL are rarely seen naturally in the field. Each of the five air sampler options listed in Table 2 was tested with six independent replicates for each starting concentration to assess repeatability and reproducibility of air samplers under the same conditions.

Prior to each trial run, the nebulizer was run for 6 min to allow for saturation of the chamber with the aerosolized C. burnetii before air sampling was started. A 30-min time gap was allowed between each run to allow sufficient time for aerosols to settle. This was followed by a thorough cleaning of the chamber, outer surfaces of the air samplers, and inner surfaces of the BSC II with 2% Virkon, 70% ethanol, and 1% sodium hypochlorite to remove any organisms and their DNA from the previous trial run. At the start of each trial run, the nebulizer was filled with 50 mL of suspension and the remaining volume was measured at the end of the trial to calculate the nebulized volume in that trial run. To avoid any bias associated with testing a given air sampler in a particular order, replicates of each air sampler option were tested in a randomized order during each trial. The collection medium or GMF was retained at the end of each trial run and stored at 4°C until further processing.

### DNA extraction and qPCR.

Prior to extraction, the GMFs were dissolved using 2 mL of UltraPure DNase/RNase-free distilled water (Thermo Fisher Scientific, USA), which had been prewarmed to 45°C. DNA was extracted from 200 μL of each liquid collection medium and dissolved GMF sample using a HiYield genomic DNA minikit (Real Biotech Corporation, Taiwan) according to the manufacturer’s protocol. Extracted DNA was tested in duplicate by qPCR targeting the single-copy *com1* gene or the multicopy IS*1111* insertion sequence (56, 57). Where the *com1* gene was targeted, C. burnetii DNA was quantified using a standard curve prepared using a synthetic control containing the *com1* assay target sequence (gBlocks gene fragment; Integrated DNA Technologies, Singapore), with one *com1* copy equivalent to one genome copy (genome equivalent [GE]). UltraPure water served as the negative control in all PCR assays. Those samples showing the typical amplification curve with a cycle threshold (*C_T_*) value below 40 were considered positive. For those samples tested by *com1* qPCR assay, percentage of recovery was calculated as

(1) $$\text{percentage of recovery}=\left[\frac{\text{GE}/\text{milliliter recovered }\times \text{ volume recovered}}{\text{GE}/\text{milliliter nebulized }\times \text{ volume nebulized}}\right]\;\times 100$$

### Determination of limit of detection, limit of quantitation, and extraction efficiency.

Prior to the sampling trials, the extraction efficiency, LoD, and LoQ of the *com1* qPCR for the three matrices used in this study (PBS, Alk PEG, and dissolved GMF) were estimated. The synthetic control was spiked at five concentrations (10^2^ GE/mL to 10^6^ GE/mL) into each of the matrices. For a given concentration of a given substrate, four DNA extractions were performed. The *com1* qPCR was performed in triplicate, resulting in 12 qPCR results for each concentration in each substrate. Acceptance criteria used in this study are summarized in Table 6.

**TABLE 6:** Parameters and acceptance criteria for validation

Parameter	Acceptance criterion
Limit of detection (LoD)	Lowest concn of *com1* target that is detected in ≥95% of replicates
Limit of quantitation (LoQ)	Lowest concn of *com1* target that can be quantified with a coefficient of variation (CV) of <25% (44)
Overall extraction efficiency	Mean of extraction efficiency at each concn above the LoQ

Estimates of the LoD and LoQs were obtained by fitting generalized linear probit models (58) to the qPCR results, using random effects to represent replicates and bootstrapping to generate confidence intervals around each LoD and LoQ estimate. For our LoQ estimations, the CV was based on the calculated C. burnetii DNA concentration (GE per milliliter) for each dilution:

(2) $$\text{coefficient of variation }=\left[\frac{\text{standard deviation}}{\text{mean}}\right]$$

Extraction efficiency was calculated as:

(3) $$\text{extraction efficiency}=\left[\frac{\text{quantified concentration}}{\text{known concentration}}\right]$$
