# Supplementary material for: Performance Evaluation and Validation of Air Samplers To Detect Aerosolized Coxiella burnetii
Source: Microbiol Spectr. 2022 Sep 8;10(5):e00655-22. doi: 10.1128/spectrum.00655-22 (PMC9602806; doi:10.1128/spectrum.00655-22)
Supplement: Supplemental file 1 — Supplemental material. Download spectrum.00655-22-s0001.pdf, PDF file, 0.7 MB [file spectrum.00655-22-s0001.pdf]

Supplementary material

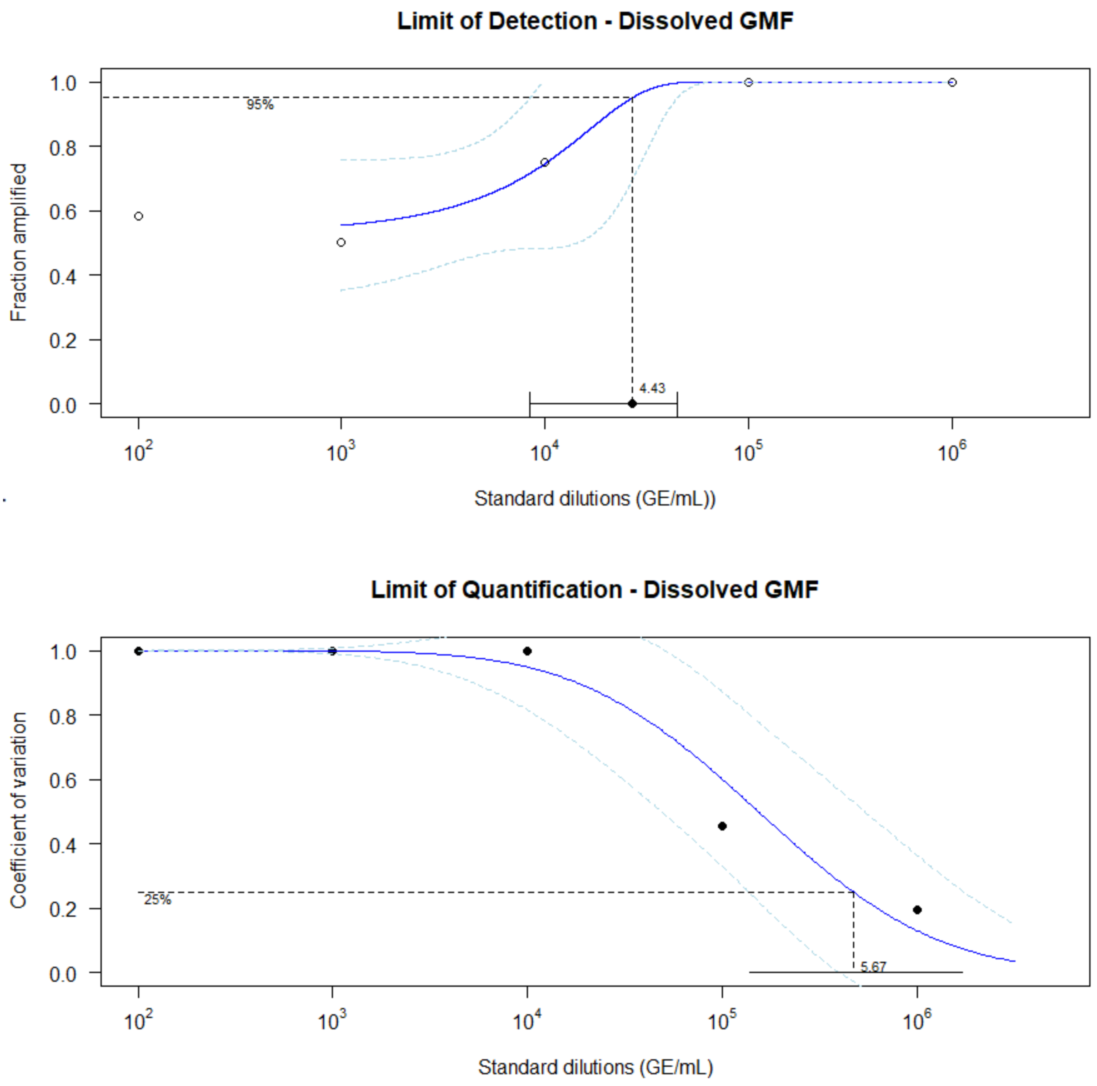

Figure S1: Limits of detection and quantitation estimates of dissolved gelatine membrane filter and their 95% confidence intervals. Solid lines depict lines of best fit while two dotted lines above and below show their 95% confidence intervals.

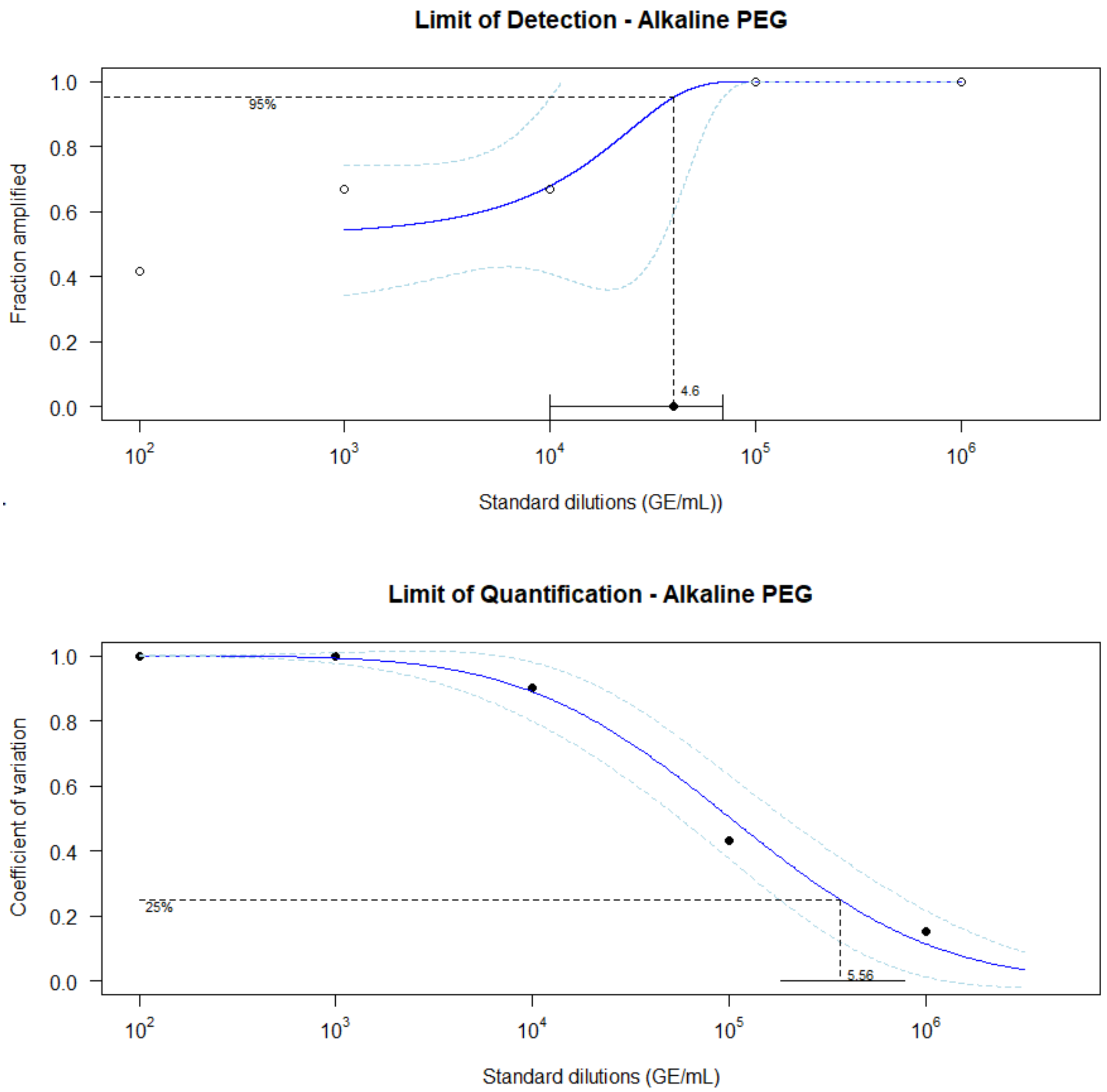

Figure S2: Limits of detection and quantitation estimates of alkaline polyethylene glycol and their 95% confidence intervals. Solid lines depict lines of best fit while two dotted lines above and below show their 95% confidence intervals.

Table S1: Detailed results of the high starting concentration (10^6 GE/mL) trial as measured by the *com1* qPCR assay.

**Air sampler**	**Nebulised volume (mL)**	**Total number of GE nebulised**	**End collection liquid volume (mL)**	**Recovered concentration (GE/mL)**	**Total number recovered**	**Percent recovery (%)**	**Mean GE/L of air †**
AirPort MD8	3.5	$10^{6.54}$	2	$10^{5.88}$	$10^{6.18}$	43.57	$10^{3.26}$
	3.5	$10^{6.54}$	2	$10^{5.31}$	$10^{5.61}$	11.57	
	3.5	$10^{6.54}$	2	$10^{5.56}$	$10^{5.86}$	20.56	
	3	$10^{6.48}$	2	$10^{5.78}$	$10^{6.08}$	40.33	
	2.5	$10^{6.40}$	2	$10^{5.65}$	$10^{5.95}$	35.70	
	2.5	$10^{6.40}$	2	$10^{5.55}$	$10^{5.85}$	28.28	
BioSampler PBS	7.5	$10^{6.88}$	7.5	$10^{4.91}$	$10^{5.79}$	8.18	$10^{2.77}$
	6.5	$10^{6.81}$	7.4	$10^{4.38}$	$10^{5.25}$	2.74	
	8	$10^{6.90}$	7.3	$10^{4.93}$	$10^{5.80}$	7.80	
	7.5	$10^{6.88}$	7.2	$10^{4.32}$	$10^{5.18}$	2.00	
	6.5	$10^{6.81}$	7.8	$10^{3.58}$	$10^{4.47}$	0.45	
	6.5	$10^{6.81}$	7.8	$10^{4.29}$	$10^{5.18}$	2.34	
BioSampler Alk PEG	8.6	$10^{6.93}$	6.9	0	0	0.00	$10^{2.25}$
	7.6	$10^{6.88}$	7.1	$10^{4.26}$	$10^{5.12}$	1.72	
	7	$10^{6.85}$	7.6	$10^{4.23}$	$10^{5.11}$	1.84	

	6.5	$10^{6.81}$	8.4	$10^{4.08}$	$10^{5.01}$	1.57	
	7	$10^{6.85}$	7.6	$10^{3.77}$	$10^{4.66}$	0.65	
	5.2	$10^{6.72}$	8.1	$10^{4.20}$	$10^{5.11}$	2.47	
Coriolis PBS	2.7	$10^{6.43}$	9.3	$10^{4.49}$	$10^{5.46}$	10.73	$10^{2.61}$
	2.6	$10^{6.41}$	9.3	$10^{4.63}$	$10^{5.60}$	15.38	
	2.2	$10^{6.34}$	9.3	$10^{4.32}$	$10^{5.28}$	8.75	
	1.5	$10^{6.18}$	9.3	$10^{4.14}$	$10^{5.11}$	8.65	
	1.2	$10^{6.08}$	9.2	$10^{4.05}$	$10^{5.01}$	8.59	
	1.5	$10^{6.18}$	9.3	$10^{4.08}$	$10^{5.04}$	7.38	
Coriolis Alk PEG	2.6	$10^{6.41}$	8.3	$10^{4.11}$	$10^{5.03}$	4.15	$10^{2.41}$
	2.8	$10^{6.45}$	8.6	$10^{4.52}$	$10^{5.45}$	10.09	
	2.5	$10^{6.40}$	8.9	$10^{3.99}$	$10^{4.94}$	3.52	
	1.6	$10^{6.20}$	8.5	$10^{3.77}$	$10^{4.70}$	3.15	
	1.2	$10^{6.08}$	8.5	$10^{4.25}$	$10^{5.18}$	12.56	
	1.2	$10^{6.08}$	8.3	$10^{4.06}$	$10^{4.98}$	7.99	

†Calculated by dividing the ‘total number recovered’ by 500 (500 L of air were collected in every run)

Table S2: Detailed results of the intermediate starting concentration (10^4 GE/mL) trial as measured by the *com1* qPCR assay.

**Air sampler**	**Nebulised volume (mL)**	**Total number of GE nebulised**	**End collection liquid volume (mL)**	**Recovered concentration (GE/mL)**	**Total number recovered**	**Percent recovery (%)**
AirPort MD8	3.5	$10^{4.54}$	2	0	0	0.00
	3.5	$10^{4.54}$	2	0	0	0.00
	3.5	$10^{4.54}$	2	$10^{3.56}$	$10^{3.86}$	20.54
	2.5	$10^{4.40}$	2	$10^{1.93}$	$10^{2.23}$	0.68
	2.5	$10^{4.40}$	2	$10^{2.40}$	$10^{2.70}$	2.00
	2.2	$10^{4.34}$	2	$10^{2.12}$	$10^{2.42}$	1.20
BioSampler PBS	8.7	$10^{4.94}$	7.2	$10^{3.46}$	$10^{4.32}$	23.79
	9.1	$10^{4.96}$	7.8	0	0	0.00
	9	$10^{4.95}$	7.7	$10^{2.92}$	$10^{3.80}$	7.08
	7.3	$10^{4.86}$	7.5	0	0	0.00
	6.8	$10^{4.83}$	7.4	$10^{1.10}$	$10^{1.97}$	0.14
	6.2	$10^{4.79}$	7.5	0	0	0.00
BioSampler Alk PEG	9.1	$10^{4.96}$	7.1	$10^{2.91}$	$10^{3.76}$	6.34
	9	$10^{4.95}$	7.4	0	0	0.00
	9	$10^{4.95}$	6.9	0	0	0.00
	7.3	$10^{4.86}$	7	0	0	0.00
	6.3	$10^{4.80}$	7.1	0	0	0.00

	6	$10^{4.78}$	6.9	0	0	0.00
Coriolis PBS	2.5	$10^{4.40}$	9.3	0	0	0.00
	1.5	$10^{4.18}$	9.3	0	0	0.00
	2.5	$10^{4.40}$	9.2	0	0	0.00
	1.6	$10^{4.20}$	9.1	0	0	0.00
	1.7	$10^{4.23}$	9	0	0	0.00
	1.5	$10^{4.18}$	9	0	0	0.00
Coriolis Alk PEG	3	$10^{4.48}$	8.3	0	0	0.00
	2.5	$10^{4.40}$	8.1	0	0	0.00
	2.5	$10^{4.40}$	8.2	$10^{2.91}$	$10^{3.82}$	26.65
	1.3	$10^{4.11}$	8.4	$10^{1.79}$	$10^{2.71}$	3.97
	1.2	$10^{4.08}$	8.1	0	0	0.00
	1.8	$10^{4.26}$	8.3	0	0	0.00

Table S3: Detailed results of the low starting concentration (10^3 GE/mL) trial as measured by the *com1* qPCR assay.

**Air sampler**	**Nebulised volume (mL)**	**Total number of GE nebulised**	**End collection liquid volume (mL)**	**Recovered concentration (GE/mL)**	**Total number recovered**	**Percent recovery (%)**
AirPort MD8	3	$10^{3.48}$	2	0	0	0
	3	$10^{3.48}$	2	0	0	0
	3	$10^{3.48}$	2	0	0	0
	2.8	$10^{3.45}$	2	0	0	0
	3	$10^{3.48}$	2	0	0	0
	3	$10^{3.48}$	2	0	0	0
BioSampler PBS	8	$10^{3.90}$	8	0	0	0
	7	$10^{3.85}$	7.6	0	0	0
	7.5	$10^{3.88}$	7.1	0	0	0
	8.3	$10^{3.92}$	7.7	0	0	0
	5.6	$10^{3.75}$	7.6	0	0	0
	6.2	$10^{3.79}$	7.6	0	0	0
BioSampler Alk PEG	7.5	$10^{3.88}$	7.2	0	0	0
	7.5	$10^{3.88}$	6.9	0	0	0
	8	$10^{3.90}$	6.5	0	0	0
	5.6	$10^{3.75}$	7.1	0	0	0
	6.5	$10^{3.81}$	7.5	0	0	0

	6.7	$10^{3.83}$	7	0	0	0
Coriolis	2.5	$10^{3.40}$	9.1	0	0	0
PBS	2.2	$10^{3.34}$	9.1	0	0	0
	2	$10^{3.30}$	9.1	0	0	0
	2.1	$10^{3.32}$	9.1	0	0	0
	1.8	$10^{3.26}$	9.2	0	0	0
	1.5	$10^{3.18}$	9.1	0	0	0
Coriolis	3.5	$10^{3.54}$	8.3	0	0	0
Alk PEG	1.5	$10^{3.18}$	8.5	0	0	0
	2.5	$10^{3.40}$	8.5	0	0	0
	1.9	$10^{3.28}$	8.3	0	0	0
	1.7	$10^{3.23}$	8.4	0	0	0
	2.1	$10^{3.32}$	8.6	0	0	0

Table S4: PCR assay validation detailed results for phosphate buffered saline.

Replicate (GE/mL)	Starting concentration (GE/mL)	Recovered concentration (GE/mL)	Extraction efficiency (%)	Mean extraction efficiency of replicates (%)	Coefficient of variation
1	10^{6}	$10^{5.50}$	31.55%		
1		$10^{5.49}$	30.75%		
1		$10^{5.56}$	36.10%	32.80%	7.18%
2		$10^{5.40}$	24.85%		
2		$10^{5.44}$	27.80%		
2		$10^{5.41}$	25.70%	26.12%	4.75%
3		$10^{5.33}$	21.35%		
3		$10^{5.25}$	17.65%		
3		$10^{5.48}$	30.30%	23.10%	22.99%
4		$10^{5.59}$	38.95%		
4		$10^{5.56}$	36.00%		
4		$10^{5.59}$	39.30%	38.08%	3.89%
overall	10^{6}	$10^{5.48}$	30.03%		21.93%
1	10^{5}	$10^{4.72}$	52.50%		
1		$10^{4.63}$	42.60%		
1		$10^{4.63}$	42.55%	45.88%	10.20%
2		$10^{4.37}$	23.50%		
2		$10^{4.53}$	33.75%		

2		$10^{4.54}$	35.05%	30.77%	16.79%
3		$10^{4.32}$	20.70%		
3		$10^{4.23}$	17.10%		
3		$10^{4.28}$	18.90%	18.90%	7.78%
4		$10^{4.11}$	12.75%		
4		$10^{4.03}$	10.65%		
4		$10^{4.14}$	13.90%	12.43%	10.82%
overall	10^5	$10^{4.43}$	27.00%		49.04%
1	10^4	0	0.00%		
1		$10^{3.16}$	14.43%		
1		$10^{2.79}$	6.18%	6.87%	86.06%
2		$10^{2.78}$	6.03%		
2		$10^{3.27}$	18.80%		
2		$10^{2.29}$	1.94%	8.92%	80.51%
3		$10^{3.17}$	14.83%		
3		0	0.00%		
3		$10^{2.25}$	1.77%	5.53%	119.56%
4		$10^{2.76}$	5.80%		
4		$10^{2.43}$	2.68%		
4		$10^{2.88}$	7.65%	5.38%	38.20%
overall	10^4	$10^{2.82}$	6.67%		89.45%
1	10^3	$10^{2.58}$	38.35%		
1		0	0.00%		

1		0	0.00%	12.78%	141.42%
2		0	0.00%		
2		$10^{3.35}$	223.50%		
2		$10^{3.10}$	127.00%	116.83%	78.34%
3		0	0.00%		
3		0	0.00%		
3		$10^{3.26}$	183.50%	61.17%	141.42%
4		$10^{4.12}$	1330.00%		
4		$10^{4.46}$	2910.00%		
4		$10^{2.57}$	37.50%	1425.83%	82.38%
overall	10^{3}	$10^{3.61}$	404.15%		206.76%
1	10^{2}	$10^{3.46}$	2905.00%		
1		$10^{3.46}$	2890.00%		
1		$10^{3.45}$	2810.00%	2868.33%	1.45%
2		0	0.00%		
2		$10^{3.93}$	8550.00%		
2		$10^{3.10}$	1255.00%	3268.33%	115.34%
3		0	0.00%		
3		$10^{2.88}$	755.00%		
3		$10^{2.76}$	575.00%	443.33%	72.63%
4		$10^{3.30}$	2005.00%		
4		0	0.00%		
4		$10^{3.24}$	1750.00%	1251.67%	71.20%
overall	10^{2}	$10^{3.29}$	1957.92%		115.48%

Table S5: PCR assay validation detailed results for alkaline polyethylene glycol.

**Replicate (GE/mL)**	**Starting concentration (GE/mL)**	**Recovered concentration (GE/mL)**	**Extraction efficiency (%)**	**Mean extraction efficiency of replicates (%)**	**Coefficient of variation**
1	10^{6}	$10^{5.41}$	25.70%		
1		$10^{5.38}$	24.25%		
1		$10^{5.34}$	21.70%	23.88%	6.92%
2		$10^{5.39}$	24.55%		
2		$10^{5.44}$	27.70%		
2		$10^{5.44}$	27.50%	26.58%	5.42%
3		$10^{5.53}$	33.50%		
3		$10^{5.49}$	30.60%		
3		$10^{5.46}$	29.00%	31.03%	6.00%
4		$10^{5.33}$	21.20%		
4		$10^{5.36}$	23.10%		
4		$10^{5.30}$	20.15%	21.48%	5.68%
overall	10^{6}	$10^{5.41}$	25.75%		15.05%
1	10^{5}	$10^{4.07}$	11.65%		
1		$10^{4.25}$	17.60%		
1		$10^{4.40}$	25.15%	18.13%	30.46%
2		$10^{4.36}$	22.75%		
2		$10^{4.56}$	36.15%		
2		$10^{4.45}$	28.15%	29.02%	18.97%

3		$10^{4.59}$	39.10%		
3		$10^{4.39}$	24.55%		
3		$10^{4.63}$	42.35%	35.33%	21.90%
4		$10^{4.20}$	15.75%		
4		$10^{4.62}$	41.30%		
4		$10^{3.88}$	7.60%	21.55%	66.62%
overall	10^5	$10^{4.42}$	26.01%		43.19%
1	10^4	$10^{2.92}$	8.40%		
1		$10^{3.45}$	28.15%		
1		$10^{3.33}$	21.55%	19.37%	42.39%
2		0	0.00%		
2		$10^{3.34}$	22.00%		
2		$10^{2.98}$	9.50%	10.50%	85.80%
3		$10^{2.85}$	7.10%		
3		0	0.00%		
3		0	0.00%	2.37%	141.42%
4		0	0.00%		
4		$10^{3.15}$	14.20%		
4		$10^{3.42}$	26.50%	13.57%	79.81%
overall	10^4	$10^{3.06}$	11.45%		90.30%
1	10^3	$10^{2.88}$	76.00%		
1		$10^{4.23}$	1705.00%		
1		0	0.00%	593.67%	132.47%

2		$10^{2.62}$	41.35%		
2		$10^{2.57}$	37.55%		
2		$10^{3.16}$	144.50%	74.47%	66.53%
3		$10^{2.65}$	44.75%		
3		$10^{4.06}$	1145.00%		
3		0	0.00%	396.58%	133.52%
4		0	0.00%		
4		0	0.00%		
4		$10^{4.18}$	1505.00%	501.67%	141.42%
overall	10^3	$10^{3.59}$	391.60%		159.39%
1	10^2	$10^{2.88}$	760.00%		
1		0	0.00%		
1		$10^{3.23}$	1685.00%	815.00%	84.54%
2		$10^{2.67}$	466.50%		
2		0	0.00%		
2		0	0.00%	155.50%	141.42%
3		$10^{2.73}$	540.00%		
3		0	0.00%		
3		$10^{4.14}$	13750.00%	4763.33%	133.49%
4		0	0.00%		
4		0	0.00%		
4		0	0.00%	0.00%	0.00%
overall	10^2	$10^{3.16}$	1433.46%		261.28%

20

Table S6: PCR assay validation detailed results for dissolved gelatine membrane filter.

Replicate (GE/mL)	Starting concentration (GE/mL)	Recovered concentration (GE/mL)	Extraction efficiency (%)	Mean extraction efficiency of replicates (%)	Coefficient of variation
1	10^{6}	$10^{5.67}$	47.20%		
1		$10^{5.64}$	44.15%		
1		$10^{5.66}$	45.25%	45.53%	2.77%
2		$10^{5.51}$	32.25%		
2		$10^{5.44}$	27.25%		
2		$10^{5.42}$	26.20%	28.57%	9.24%
3		$10^{5.51}$	32.40%		
3		$10^{5.48}$	30.30%		
3		$10^{5.48}$	30.50%	31.07%	3.05%
4		$10^{5.63}$	42.70%		
4		$10^{5.58}$	38.30%		
4		$10^{5.59}$	39.20%	40.07%	4.74%
overall	10^{6}	$10^{5.56}$	36.31%		19.46%
1	10^{5}	$10^{4.24}$	17.20%		
1		$10^{4.12}$	13.15%		
1		$10^{4.31}$	20.60%	16.98%	17.93%
2		$10^{4.49}$	30.85%		
2		$10^{4.44}$	27.80%		
2		$10^{4.39}$	24.35%	27.67%	9.60%

3		$10^{4.80}$	63.50%		
3		$10^{4.70}$	49.60%		
3		$10^{4.77}$	58.50%	57.20%	10.05%
4		$10^{4.59}$	38.55%		
4		$10^{4.48}$	30.35%		
4		$10^{4.47}$	29.30%	32.73%	12.63%
overall	10^5	$10^{4.53}$	33.65%		45.45%
1	10^4	$10^{3.26}$	18.40%		
1		$10^{3.51}$	32.10%		
1		$10^{3.86}$	72.00%	40.83%	55.68%
2		$10^{3.59}$	39.15%		
2		$10^{2.92}$	8.30%		
2		$10^{3.78}$	60.00%	35.82%	59.30%
3		$10^{3.74}$	55.50%		
3		$10^{4.22}$	166.50%		
3		0	0.00%	74.00%	93.54%
4		0	0.00%		
4		0	0.00%		
4		$10^{2.94}$	8.65%	2.88%	141.42%
overall	10^4	$10^{3.58}$	38.38%		118.80%
1	10^3	$10^{4.13}$	1360.00%		
1		$10^{2.83}$	67.50%		
1		$10^{3.45}$	279.00%	568.83%	99.51%

2		0	0.00%		
2		$10^{3.99}$	985.00%		
2		0	0.00%	328.33%	141.42%
3		$10^{2.91}$	82.00%		
3		0	0.00%		
3		0	0.00%	27.33%	141.42%
4		0	0.00%		
4		0	0.00%		
4		$10^{2.70}$	50.50%	16.83%	141.42%
overall	10^{3}	$10^{3.37}$	235.33%		183.87%
1	10^{2}	0	0.00%		
1		$10^{3.13}$	1335.00%		
1		$10^{2.86}$	730.00%	688.33%	79.29%
2		0	0.00%		
2		0	0.00%		
2		$10^{3.60}$	3975.00%	1325.00%	141.42%
3		$10^{3.64}$	4415.00%		
3		$10^{3.68}$	4750.00%		
3		0	0.00%	3055.00%	70.85%
4		0	0.00%		
4		$10^{3.25}$	1775.00%		
4		$10^{3.88}$	7600.00%	3125.00%	103.88%
overall	10^{2}	$10^{3.31}$	2048.33%		118.53%
